# The Management of Sarcopenic Dysphagia: A Multidisciplinary Approach Leveraging Emerging Technologies

**DOI:** 10.14336/AD.2024.0741

**Published:** 2025-09-26

**Authors:** Wei Deng, Xiao-Feng Zeng, Li-Ying Zhang, Ji-Rong Yue, Xiao-Li Huang

**Affiliations:** The Center of Gerontology and Geriatrics, National Clinical Research Center for Geriatrics, West China Hospital, Sichuan University, Chengdu, Sichuan, China

**Keywords:** aging, sarcopenic dysphagia, multidisciplinary care, technology, advancements

## Abstract

With the continuous improvement of people's understanding of healthy aging, how to age decently and healthily has become a new topic of human exploration. Various factors make sarcopenia and dysphagia very common in the elderly, and the concept of "sarcopenic dysphagia" was born. This review synthesizes and extends the current understanding of sarcopenic dysphagia, emphasizing novel diagnostic and therapeutic strategies, particularly emerging technologies and multidisciplinary approaches. We begin by discussing pathophysiology and overlap with malnutrition and oral frailty. Next, we explore cutting-edge diagnostic tools and treatment modalities, focusing on how recent technological advancements have reshaped clinical practices. This review highlights the significance of integrating care across specialties, including nutrition, physical therapy, and speech-language pathology, to offer holistic care. It is hoped that our review will contribute to further understanding of sarcopenic dysphagia and thus provide new insights into promoting healthy aging in the elderly.

## 1. Introduction

### 1.1 Emerging Challenges in an Aging Population

Over the last few decades, global attention has increasingly turned toward the rapid aging of populations. Elderly refers to people aged 65 years and older. According to the "World Population Aging 2023" by the United Nations, population aging is a problem in almost all regions and areas of the world. The proportion of the elderly and the total elderly population will vary across different regions and are expected to continue increasing in the upcoming decades [1]. Unfortunately, prolonged life expectancy has not been met with a corresponding decrease in chronic diseases.

Consequently, this longevity paradox manifests in poorer health outcomes for elderly individuals, imposing substantial burdens on families and society. The significance of aging research has surged, given the escalating demand for improved quality of life and healthier aging experiences. The health and functional trajectories of older adults throughout their life course are intricately linked to multiple factors, which include their genetic and environmental backgrounds and various physiological and psychological factors [2]. Notably, a common denominator among older individuals is the gradual decline in several physiological functions, heightening susceptibility to conditions such as sarcopenia, dysphagia, osteoporosis, and frailty in older people [3-6].

### 1.2 Overview of sarcopenic dysphagia

Sarcopenia is a pathological condition characterized by a progressive and widespread reduction in skeletal muscle mass and a corresponding decrease in muscle strength. This condition typically initiates between the ages of 40 and 50 and impacts 9.9% and 40.4% of the older population [7].

Dysphagia is a difficulty in eating and swallowing and is characterized by impaired chewing and swallowing, inability to reach, or prolonged transit of food or fluids from the oral cavity to the esophagus [8]. The oropharyngeal swallowing process is a coordinated set of neuromuscular actions, typically divided into four distinct stages, namely, the oral preparatory, oral, pharyngeal, and esophageal phases, which allow the delivery of bolus food from the oral cavity to the upper esophageal sphincter [9]. Therefore, oropharyngeal dysphagia, which is difficulty swallowing due to poor oral intake, may lead to dehydration and malnutrition, increasing the risk of muscle loss [8].

**Figure 1.**
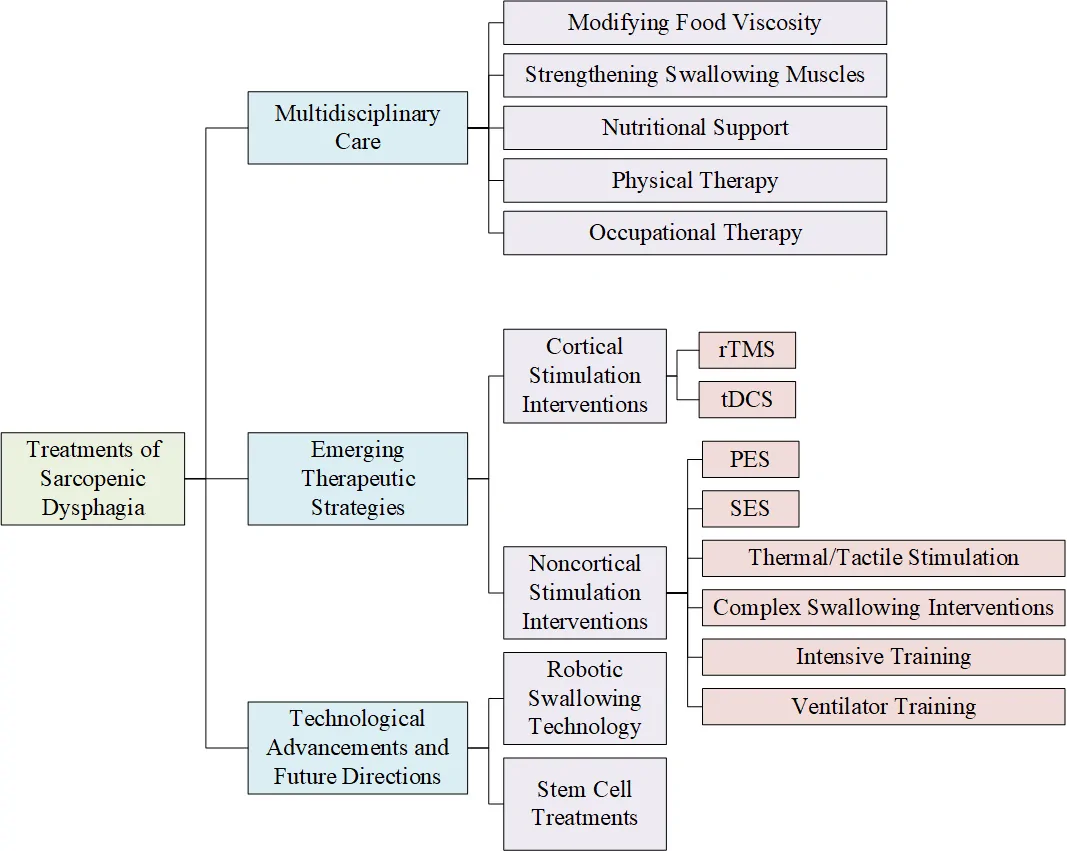
Multidisciplinary therapeutic strategies of sarcopenic dysphagia.

Moreover, because sarcopenia reduces the strength of swallowing muscles [3, 10, 11], it may also be accompanied by changes in swallowing function. This resulted in the concept of "sarcopenic dysphagia," which is a swallowing disorder caused by a decrease in systemic skeletal muscles and swallowing muscles. Swallowing dysfunction may be aggravated by sarcopenia, which is currently a swallowing disorder of great concern in the medical community. Dysphagia without systemic sarcopenia cannot be considered sarcopenic dysphagia [12, 13]. Given the detrimental impact of sarcopenia on the Health-Related Quality of Life (HRQoL) and physical capabilities of older people, the importance of embracing a multidisciplinary rehabilitation strategy alongside effective nutritional management cannot be ignored [14, 15]. Nowadays, there are more and more therapeutic strategies for sarcopenic dysphagia (Fig. 1).

## 2. Sarcopenic dysphagia, Malnutrition and Oral frailty

The oral cavity, as the initial part of the digestive system, serves various functions, such as biting, chewing, and aiding in bolus formation [16]. Studies have revealed associations between poor oral health and inadequate diet in older adults, increasing malnutrition risk [17]. Tooth loss impacts food choices and nutritional status in elderly people [18, 19]. A 2021 review revealed that edentulous individuals had a 9.5% greater risk of malnutrition [20]. Oral frailty, encompassing factors such as remaining teeth and masticatory performance, is correlated with severe malnutrition [21]. Additionally, oral dryness, or xerostomia, prevalent in elderly individuals, is linked to medication use and autoimmune conditions, affecting overall oral health and quality of life [22-24]. Reduced salivary flow in older individuals leads to various oral issues and increases the risk of infections [25-29]. Periodontal disease, a gum inflammation, can lead to tooth loss and impact systemic health [30-34]. Nutritional deficiencies contribute to immune system impairment [35, 36]. Poor oral health is associated with chronic inflammation, potentially leading to weight loss and muscle depletion [37].

The link between oral health, dysphagia, sarcopenia, and malnutrition suggests a detrimental cycle. It shows the importance of addressing oral health for overall well-being in older individuals.

## 3. Innovations in Diagnosis

### 3.1 Recent advancements in diagnostic tools

Sarcopenic dysphagia is a swallowing disorder caused by a reduction in skeletal and swallowing muscle mass [38]. The diagnostic criteria for classifying subjects into three categories, confirmed sarcopenic dysphagia, probable sarcopenic dysphagia, and nonsarcopenic dysphagia, involve five steps [39]:

1. Presence of swallowing difficulty;
2. Existence of systemic muscle reduction;
3. Imaging results consistent with the loss of swallowing muscle mass (such as computed tomography (CT), magnetic resonance imaging (MRI), and ultrasound);
4. Exclusion of other causes of dysphagia beyond muscle reduction;
5. Muscle reduction is a primary cause of dysphagia, but other causes, such as stroke, brain injury, neuromuscular diseases, head and neck cancer, and connective tissue diseases, may also exist.

A definite diagnosis can be made if the first four criteria are met; a high suspicion arises with criteria 1, 2, and 4; and a possible diagnosis can be made with criteria 1, 2, and 5. It is worth noting that many elderly patients often fall into the latter category [40].

Notably, these standards do not include a direct assessment of swallowing strength. Although measuring tongue pressure or head lift strength is suggested to check for a decrease in tongue muscle strength, which could indicate sarcopenic dysphagia, the third criterion, involving imaging of swallowing muscles, is challenging to implement in a clinical routine focusing on functional assessments. Moreover, the lack of clear, clinically relevant operational standards for swallowing muscle mass loss makes this controversial. These challenges limit the widespread clinical application of sarcopenia definitions, particularly the need for a greater association between body composition assessment and clinical practice and the absence of widely accepted thresholds for muscle mass differences.

The assessment of sarcopenia includes muscle mass, muscle strength, and physical performance [41, 42]. Currently, various validated clinical methods and instruments are recommended for evaluating sarcopenia. These methods include parameters suggested by the 2019 Asian Working Group for Sarcopenia (AWGS), such as the SARC-F, SARC-CalF, Calf Circumference (CC), Appendicular Skeletal Muscle Index (ASMI), handgrip strength (HGS), gait speed (GS), Short Physical Performance Battery (SPPB), and 5-Time Sit To Stand Test(5TSTS) [38]. Muscle mass assessment primarily relies on dual-energy X-ray absorptiometry (DXA) and Bioelectrical Impedance Analysis (BIA), while muscle strength measurement focuses on handgrip strength using dynamometers. Physical function assessment included tests such as the 6-Minute Walk Test (6MWT), SPPB scoring, 5TSTS, Timed Up and Go Test (TUG), and 400-Meter Walk Test (400MWT).

Recent advancements in diagnostic tools for sarcopenic dysphagia have focused on the development of reliable and valid algorithms [40], as well as the use of various measures such as handgrip strength, skeletal muscle mass/index, tongue pressure, gait speed, and calf circumference [43]. Imaging techniques, including DXA, CT, MRI, and ultrasound, have also been explored for the diagnosis of sarcopenia, a key component of sarcopenic dysphagia [44]. Ultrasound, in particular, has shown promise in differentiating age-related muscular features [45]. However, further research is needed to validate these diagnostic tools and establish cutoff values for different populations.

### 3.2 Technology-driven approaches

Imaging techniques enhance diagnostic accuracy and provide a comprehensive understanding of muscle composition, facilitating early identification and management of sarcopenic dysphagia.

#### DXA

DXA is a radio diagnostic method most commonly used in research and clinical practice because it is simple, accessible, minimally ionizing radiation exposure [41], highly accurate, repeatable, and cost-effective [46]. According to the European Working Group on Sarcopenia(EWGS) in Older People, DXA is considered a reliable tool and the most common method for measuring body composition, providing quantitative data on the distribution of lean, fat, and mineral contents of the body [41] and offering a low-dose option for assessing body composition.

#### CT/MRI

Tomographic imaging techniques, such as CT and MRI, are considered the gold standard for diagnosing sarcopenia because both methods can accurately assess body composition and the consistency of skeletal muscles in cross-sectional images [45]. Giraudo [47] highlighted the role of various radiological techniques, such as MRI and CT, in assessing muscle loss, a key component of sarcopenic dysphagia. These techniques provide robust qualitative and quantitative information, aiding in the early and accurate diagnosis of the condition. MRI includes magnetic resonance spectroscopy (MRS) for noninvasive molecular analysis of muscle tissue [47]. CT can be used for other indications to obtain additional parameters of skeletal muscles (opportunistic use of CT data), such as total abdominal muscle area (TAMA) or indices for lumbar and pectoral muscles [48-52]. CT is also of great value in prognosis prediction and rehabilitation guidance for elderly patients with sarcopenic dysphagia. Head and neck cancer patients before treatment with less muscle disease, are more likely to have difficulty swallowing before the therapy and breathing after treatment. Sarcopenia assessment by CT can help identify patients at high risk of swallowing dysfunction and provide them with interventions such as pre-rehabilitation [53]. In oropharyngeal cancer patients during treatment, less muscle disease can also occur through regular head and neck CT evaluation, studies have shown that patients with sarcopenia are significantly more likely to place the feeding tube, conducive to the management of patients with oropharyngeal cancer patients with nutritional support, and to predict the survival of the results [53, 54]. Besides, combined with a CT image early identification of sarcopenic dysphagia can help stroke patients with swallowing disorder risk stratification, more accurate diet advice, and swallowing training plans. Training targeting swallowing muscles has been confirmed to improve dysphagia after stroke [55]. However, despite their high diagnostic value, the use of CT and MRI is limited by the high ionizing radiation burden or specific contraindications, respectively, and is restricted by high costs and postprocessing image analysis; therefore, their use is typically confined to research settings [45].

#### Ultrasound

Ultrasound is a cost-effective, widely used, and radiation-free method with excellent correlation to MRI measurements, offering a good alternative with high repeatability [56]. Ultrasound is an excellent imaging modality for assessing the musculoskeletal system [57], in particular, and has shown promise in differentiating age-related muscular features [45]. However, Its use in evaluating patients with sarcopenia is relatively limited to assessing muscle thickness, cross-sectional area, and echogenicity [45]. Currently, no diagnostic algorithm includes ultrasound as one of the possible diagnostic methods for assessing sarcopenia, possibly because most ultrasound parameters depend on the operator, and some cannot fully differentiate the minimal changes in muscle condition [44]. However, there are more and more researchers who see the value of ultrasonic diagnosis of sarcopenic dysphagia. With the development of ultrasound technology and the continuous in-depth study of ultrasound in the diagnosis of sarcopenia or sarcopenic dysphagia, ultrasound imaging of swallowing-related muscles (geniohyoid muscle, tongue muscle, digastric muscle) has also been paid more and more attention by researchers [58]. Mori's study used ultrasound to measure the effectiveness of the cross-sectional area of geniohyoid muscle and proposed the standard and threshold for the Asian population. The application significance of the cut-off values is that they can be used to diagnose sarcopenic dysphagia, especially in the elderly female population, with parameters of sarcopenia. By incorporating these cut-off values, the new diagnostic algorithm can be further improved for more effective use in clinical practice [59].

## 4. Traditional Treatment Methods

Traditional treatments for sarcopenic dysphagia can improve the patient's symptoms through changes in food texture, proper nutritional support, exercise training, pharmacological interventions, surgery, and denture fabrication.

### 4.1 Texture-Modified Diets (TMDs)

In patients with sarcopenic dysphagia, food texture modification is required to ensure a safe swallowing process. The International Dysphagia Diet Standardisation Initiative (IDDSI) guideline in 2015 classified different types of TMDs into nine levels, with liquids classified as grades 0 to 4, and foods classified as grades 3 to 7. Grade 0 is for dilute liquids (e.g., water); the thickness of the liquid increases as the grade increases, with grade 4 representing extremely thick liquids. Levels 3 and 4 can also be used to describe liquefied and pureed foods, respectively. As the level increases, the particle size of the food increases, and the processing required decreases. level 5 represents chopped and moist; level 6, soft, bite-sized; level 7EC, easy to chew; and level 7 represents regular food. The guidelines contain standardized definitions and descriptions of different levels of dysphagia diets [60].

TMDs are meal forms designed to assist patients with limited chewing and/or swallowing ability. However, because TMDs lose some nutrients through special cooking methods and the addition of liquids, it is necessary to provide energy- and protein-enhanced TMDs to older adults who need them to avoid loss of skeletal muscle mass [61-63].

TMDs can also be provided to patients at risk of dysphagia to prevent or delay the onset of sarcopenic dysphagia. Postural maneuvers and hygienic and dietetic rules also play a positive role in the prevention of sarcopenic dysphagia [64].

### 4.2 Nutritional Support

Appropriate nutritional support may significantly improve nutrient intake in patients with sarcopenic dysphagia and may also be effective in improving tongue muscle strength in patients with sarcopenic dysphagia. Active nutritional therapy is an approach to nutritional management that utilizes the patient's daily energy expenditure and daily energy accumulation to establish daily energy requirements, thereby increasing the patient's body weight and muscle mass. A weight gain of 1kg requires approximately 7500 kcal. An energy intake of ≥30 kcal/kg/day and a protein intake of ≥1.2 g/kg/day based on ideal body weight have a significant impact on the increase in tongue strength in elderly patients with sarcopenia [65]. Nutritional support program includes protein supplemented diet, supplementation with specific amino acids, α-linoleic acid, β-hydroxy-β-butyric acid methyl ester, vitamin D, etc.

### 4.3 Exercise Training

Exercise training can improve physical mobility and increase muscle capacity in older adults. The therapeutic intervention for sarcopenic dysphagia, on the other hand, focuses on improving the strength and function of swallowing-related muscles. Various exercises and maneuvers, such as Shaker exercise and related exercises, Mendelsohn maneuver, and Masako maneuver are used to improve swallowing function. In addition, swallowing muscle strengthening exercises such as chin tuck against resistance(CTAR) exercises, resistive jaw-opening exercise (RJOE) exercises, swallow resistance exercises, tongue pressure resistance training (TPRT), and lingual exercises can improve swallowing in patients with sarcopenic dysphagia [66].

### 4.4 Pharmacological Interventions

Although there are currently no specific drugs approved for the treatment of sarcopenia, a variety of pharmacological interventions have been identified for improving sarcopenia in older adults, including growth hormone, growth hormone-releasing hormone, vitamin D, dehydroepiandrosterone, combined estrogen and progesterone, testosterone-growth hormone, pioglitazone, testosterone, insulin-like growth factor-1, and angiotensin-converting enzyme inhibitors [67, 68].

Possible drugs for sarcopenia are under development. Rolland's systematic review of drug trials in different clinical elderly populations (e.g., men with low gonadotropin secretion, and postmenopausal women) confirmed that the current drugs available for the treatment of sarcopenia in the elderly are limited, and the main recommendations are strength training and nutritional support [69].

### 4.5 Surgery

In patients with sarcopenic dysphagia, not all of them can improve their symptoms with swallowing therapy and dietary modifications; therefore, surgical procedures to improve swallowing function are necessary as a complement to non-surgical interventions.

**Figure 2.**
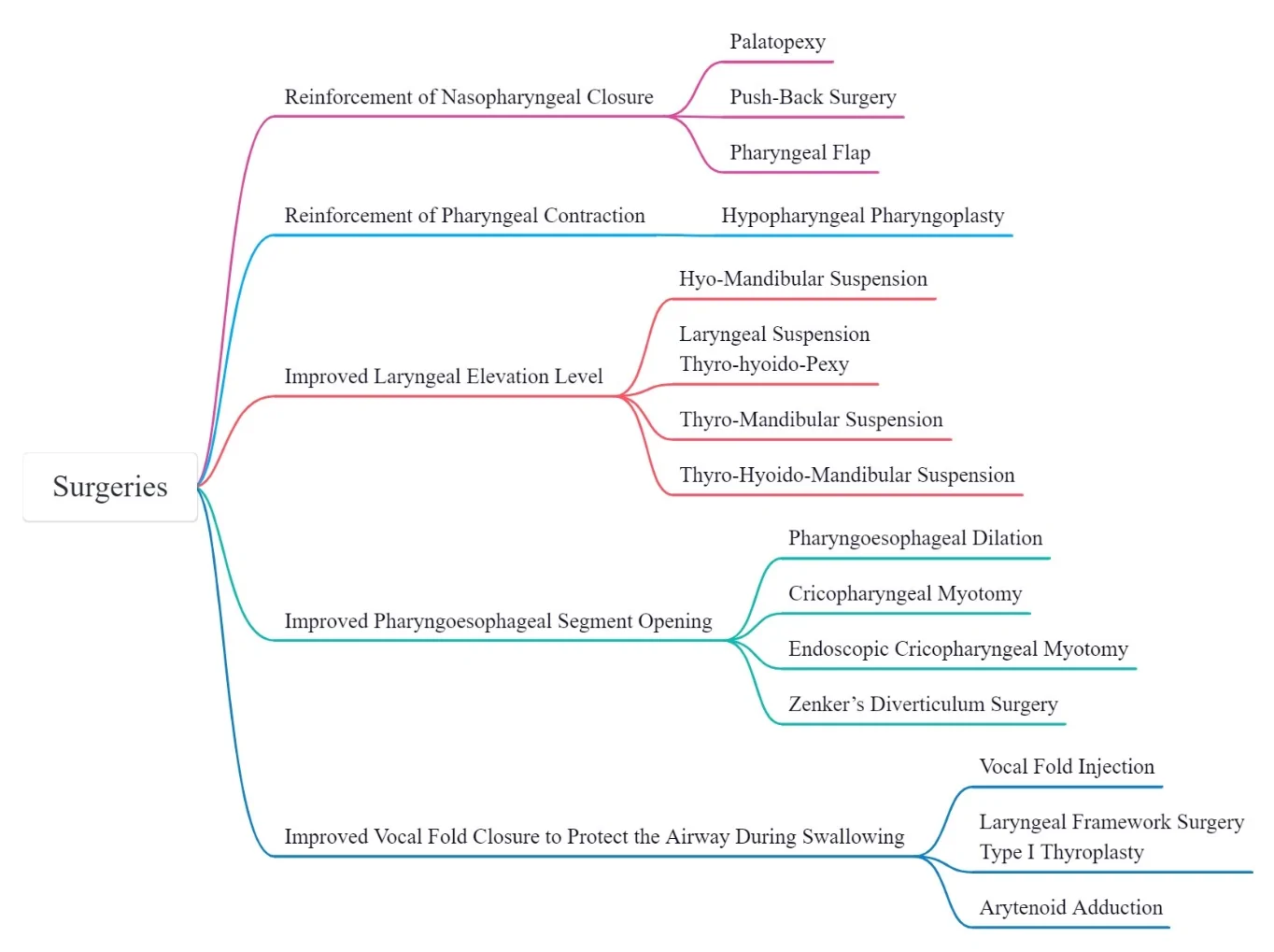
Several surgical procedures to improve swallowing function.

Surgeries to improve swallowing function involve specific dysfunctional areas of the swallowing mechanism and can be categorized as procedures to enhance nasopharyngeal closure or pharyngeal constriction, to improve laryngeal elevation or opening of the pharyngeal-oesophageal segments, and to improve vocal fold closure to protect the swallowing airway. These surgeries encompass several surgical procedures, as shown in Figure 2 [70].

### 4.6 Denture Fabrication

Wearing a denture can have a positive effect on swallowing in both the oral cavity and pharynx. The altered morphology of the pharynx in patients with dysphagia when not wearing a denture can lead to increased tongue residue, increased pharyngeal area, and prolonged passage time in the oropharyngeal segment, increasing the risk of aspiration and pneumonia in older adults [71]. In addition, the number of teeth present and tooth contact pairs, natural or artificial, significantly affect the maintenance of food form when food is divided into normal and dysphagic diets, and wearing a denture contributes to the maintenance of food form, as well as improves the functional efficiency of mastication compared to not wearing a denture [72]. When missing teeth, we encourage older adults to wear dentures for occlusal support, to improve nutritional status and activities of daily living by improving swallowing function [73].

Surgery is an effective alternative treatment that can significantly improve the quality of life of patients with sarcopenic dysphagia.

## 5. Multidisciplinary Care

### 5.1 Integrating nutrition, physical therapy, and speech-language pathology

The treatment and care of sarcopenic dysphagia require the collaborative strategy of a multidisciplinary team, and cooperation between healthcare providers and patients' families/caregivers is crucial for successful treatment [74]. Dellis emphasized the importance of multidisciplinary therapeutic interventions, which have enhanced new concepts and technologies [75]. Interventions aimed at breaking the vicious cycle between dysphagia and malnutrition include modifying food viscosity, strengthening swallowing muscles, nutritional support, physical therapy, and occupational therapy, which are the main strategies for treating sarcopenic dysphagia.

There is a comprehensive treatment plan for patients with sarcopenic dysphagia [74, 76, 77]. Modifying the viscosity of food can reduce the risk of aspiration and should be adapted to swallowing impairments. Nutritional interventions, including a high-protein diet [65] and supplementation with specific nutrients such as vitamin D [78, 79] and ω-3 fatty acids, can significantly improve the intake of macronutrients and micronutrients [80, 81], which have been proven to help increase muscle mass. Strengthening exercises for the swallowing muscles can aid in the recovery of tongue muscle strength and the practice of oropharyngeal swallowing, potentially improving swallowing function by enhancing muscle strength and endurance. Combining targeted swallowing training with nutritional interventions can improve patients’ swallowing function and quality of life [82, 83]. Physical and occupational therapies can assist in alleviating the symptoms of sarcopenia [82, 84].

### 5.2 Multidisciplinary Success Case Studies

The rehabilitation program for sarcopenic dysphagia should include two main parts: the treatment of sarcopenia and the rehabilitation of dysphagia. This requires nutritional management, physical training, general condition improvement, swallowing rehabilitation training, and various physical therapies. Therefore, the treatment of sarcopenic dysphagia relies on a variety of multidisciplinary, coordinated, comprehensive treatment programs.

Here, we show several cases in which an integrated management strategy, including aggressive nutritional support, rehabilitation training, and comprehensive nursing interventions, significantly improved sarcopenia.

An 80-year-old woman with severe dysphagia who was dependent on tube feeding did not improve with usual physical therapy or treatment for dysphagia. The patient's condition significantly improved with comprehensive intervention from a multidisciplinary nutrition support team (NST). The NST consists of a range of professionals including doctors, dieticians, nurses, dental hygienists, speech therapists, and social workers. They collaborate to provide patients with a multidisciplinary approach that includes nutritional support, physiotherapy, swallowing rehabilitation training, and active care. Interventions included enhanced nutritional intake, physical therapy, and specialized dysphagia rehabilitation training. The main aim of the NST is to improve the patient's physical and swallowing function by improving nutritional intake in conjunction with rehabilitation training. This case highlights the effectiveness of a multidisciplinary approach in the management of sarcopenic dysphagia. This highlights the importance of providing solutions to nutritional and rehabilitation needs to improve patient outcomes [84].

Another patient, a 71-year-old man, presented with sarcopenicdysphagia after lung cancer surgery. On postoperative day 16, he was referred to the Rehabilitation Medicine Department for physical and speech therapy. Physical therapy includes resistance training, movement exercises, and ambulation exercises. Speech therapy includes direct exercises using modified food and indirect exercises such as stretch and resistance training of the swallowing muscles. On postoperative day 74, the rehabilitation team predicted that the dysphagia would worsen if his nutritional status was not improved, so a percutaneous endoscopic gastrostomy was performed. On postoperative day 80, he was transferred to a NST consisting of doctors, nurses, registered dietitians, pharmacists, clinical technologists, physical therapists, occupational therapists, and a speech therapist for more systematic and professional rehabilitation training and nutritional support. Oral dietary supplements and outpatient rehabilitation were continued after discharge. Although the patient initially experienced severe dysphagia and malnutrition, it improved significantly after rehabilitation, nutritional therapy, dysphagia rehabilitation, and intensive nutritional support provided by a multidisciplinary team. The patient's dysphagia improved from FOIS level 1 (unable to eat orally) to FOIS level 7 (total oral diet with no restrictions), and both physiotherapy and speech therapy were discontinued. Although still diagnosed with sarcopenia, the patient was no longer malnourished and dysphagic. This comprehensive therapy improved the patient's swallowing function and nutritional status, highlighting the effectiveness of rehabilitation and nutritional care in the treatment of sarcopenic dysphagia [85].

Moreover, a 72-year-old man with Parkinson's disease and sarcopenic dysphagia significantly improved after family-based comprehensive rehabilitation and active nutritional management after discharge. The rehabilitation training mainly included neck muscle training, oral motor training, and daily activity training. The family members or patients completed the training independently, and the training was about one hour per day for four months. Nutritional support was gradually increased from 1200 to 2800 kcal per day to help the patient regain weight and muscle mass. The nutrition program was adjusted with regular dietary records and follow-up assessments, with the goal of returning the patient to 90% of the ideal body weight. Home therapy offers several advantages over a hospital setting. First, patients can flexibly adjust the mealtime according to their state, avoiding the fixed mealtime in the hospital. Second, patients can choose familiar foods according to their personal preferences, which improves motivation and energy intake. In addition, the involvement of the family allowed the treatment plan to be implemented smoothly, and the nutritional intake and rehabilitation training were carried out as planned. Although the patient initially presented with severe dysphagia and malnutrition, he recovered normal swallowing function, gained weight, and improved muscle mass, strength, and physical function after the intervention, highlighting the effectiveness of combining rehabilitation with nutritional support in the treatment of sarcopenic dysphagia, especially in home care. Although the treatment and monitoring performed in the home may be less comprehensive than in the hospital, the combination of rehabilitation training and active dietary management in the home environment may have a good therapeutic effect on some patients. It is of great significance in improving the quality of life of patients and reducing the length of hospital stay [86].

### 5.3 Unsuccessful Cases

Early screening of swallowing function is particularly necessary for high-risk populations who may develop sarcopenic dysphagia. A detailed assessment of swallowing function and follow-up should be performed even when the patient exhibits only mild aspiration symptoms. In addition, the use of assessment tools such as bioimpedance analysis and handgrip strength testing to detect the potential risk of sarcopenic dysphagia may help to define the specific causes of dysphagia and guide clinical management. Beom first reported the rapid onset and worsening of dysphagia in patients with mandatory spondylitis within a few months after spinal cord injury, leading to severe aspiration pneumonia and sepsis, which also hinted at the possibility of multiple factors. In recent years, with the introduction of the concept of "sarcopenic dysphagia", Beom also speculated that the patient's prolonged bed rest led to muscle atrophy, which aggravated the situation of dysphagia [87].

A case report study by Buşra Can is the first to report sarcopenic dysphagia in non-intubated COVID-19 patients. The inflammatory state of COVID-19, combined with malnutrition and low activity during hospitalization, may lead to secondary sarcopenia and resultant dysphagia. This report highlights the unanticipated complications that COVID-19 infection can cause in elderly patients, particularly in the context of malnutrition and sarcopenia, where dysphagia may emerge as a major new health problem. This represents a new challenge for clinicians in the management of COVID-19 patients. [88].

Sarcopenia caused by prolonged bed rest, malnutrition, and aspiration pneumonia can lead to the occurrence of secondary dysphagia in patients, which also reminds healthcare professionals that assessment of swallowing function should be part of the routine examination of patients at risk of sarcopenic dysphagia in order to prevent the occurrence of sarcopenic dysphagia and to minimize the adverse consequences once it occurs.

## 6. Emerging Therapeutic Strategies

### 6.1 Novel treatment modalities

Several adjunctive treatment options potentially improve dysphagia recovery, and these interventions are mainly divided into cortical stimulation interventions and noncortical stimulation interventions [89]. Cortical stimulation interventions, including repetitive peripheral magnetic stimulation (rPMS), repetitive transcranial magnetic stimulation (rTMS) and transcranial direct current stimulation (tDCS), enhance weakened oropharyngeal musculature through the peripheral neuromuscular system. Noncortical stimulation intervention increases the sensory input of the brain swallowing network, increases the activity of the cortex, neural network, and motor swallowing area in the brainstem, and promotes neural plasticity after stroke to improve swallowing treatment [90]. These include neuromuscular electrical stimulation (NMES), pharyngeal electrical stimulation (PES), sensory stimulation (SES), thermal/tactile stimulation), complex swallowing interventions, intensive training, and respiratory muscle training [91].

Cortical stimulation interventions, which aim to stimulate the cerebral cortex and subcortical swallowing network directly, include rTMS and tDCS. rTMS modulates cortical excitability by locally stimulating cortical regions. tDCS is a noninvasive cortical stimulation technique designed to restore swallowing function by expanding the pharyngeal representation of the undamaged hemisphere, hypothetically ensuring increased input to the brainstem swallowing center. The present stimulation protocol was designed to facilitate this process in patients with hemispheric lesions without brainstem damage.

Compared with other treatment options, NMES is the least expensive and easiest to apply. Recently, the effectiveness of NMES and repetitive transcranial magnetic stimulation for dysphagia after stroke has been reported [92].

### 6.1.1 NMES

NMES has become an essential topic in the field of dysphagia rehabilitation. In NMES therapy, electrodes are applied to the suprahyoid or infrahyoid muscles to activate the muscles by applying current to the throat muscles, and NMES of the suprahyoid muscle has shown positive effects in the treatment of stroke and Parkinson's disease [93, 94]. In addition, NEMS of the infrahyoid muscle is also considered a swallowing resistance movement, which can be applied to patients who have difficulty performing voluntary movements to improve their swallowing function [95, 96]. The suprahyoid muscle group included the digastric, geniohyoid, mylohyoid, and stylohyoid muscles. The infrahyoid muscle group includes the sternohyoid muscle, omohyoid muscle, thyrohyoid muscle, and sternothyroid muscle.

NMES treatment of the digastric or thyrohyoid muscle can restore normal swallowing function in 35% of patients with severe stroke-induced dysphagia [97]. However, selective stimulation of the thyrohyoid muscle by surface electrodes appears to be more difficult due to the overlap between the thyrohyoid muscle and sternohyoid muscle [98]. It has been reported that 20 of 23 patients with moderate to severe dysphagia showed significant improvement after NMES of the thyrohyoid muscle during swallowing [99].

### 6.1.2 rPMS

Sometimes, NMES of the suprahyoid muscle may trigger pain in patients with sarcopenic dysphagia and prevent effective contraction of the muscle. In addition, the skin preparation process for surface NMES is time-consuming. A feasible solution is to use the rPMS technique to treat the suprahyoid muscle [66]. The rPMS technique does not activate nociceptors in the skin and can provide high-intensity stimulation with low pain [100, 101]. With rPMS, the coil does not need direct contact with the skin, thus avoiding skin preparation, including shaving, and simplifying the treatment process [66].

### 6.1.3 PES

As a noncortical stimulation method, PES has shown potential effectiveness in many studies and has become a new method of treatment. This technique targets the peripheral neuromuscular system and aims to enhance and restore the function of the damaged oropharyngeal musculature [102-105].

### 6.1.4 SES

Such interventions have been classified as noncortical, and their potential benefits in improving swallowing dysfunction are under active exploration. It has been proposed that SES may promote neuroplastic changes in the sensory cortex, although the mechanism of this action is not fully understood [106].

These findings suggest a growing interest in and development of various therapeutic approaches to sarcopenic dysphagia, especially in the context of rehabilitation after a neurological injury such as a stroke. These studies highlight the need for more standardized treatment protocols and outcome measures to better compare different interventions and enhance the overall understanding of their effectiveness.

## 6.2 Role of technology in treatment

### 6.2.1 NMES

VitalStim and the Ampcare Effective Swallowing Protocol (ESPTM) are commercial products of the NMES for patients with dysphagia [66]. The use of commercially available devices such as the VitalStim stimulator has been the focus of discussion in the clinical and research communities.

VitalStim therapy, a treatment for dysphagia that involves NMES of the swallowing muscles in the neck, was approved by the Food and Drug Administration (FDA) in 2001. The approval is based on data collected from more than 800 treated patients by the device's lead developer and patent holder. VitalStim therapy is widely recognized on the market. It successfully restored long-term swallowing function in 97.5% of patients, which significantly exceeded the need for feeding tube dependence and showed significant advantages over existing therapies [107]. It can effectively improve swallowing, neurological, and limb motor functions, reduce complications, promote physical recovery, and improve overall quality of life of patients [108].

Although VitalStim therapy has been used successfully in other parts of the world, VitalStim therapy is rarely used in Pakistan. As reported by the Speech and Language Therapy Department of Ziauddin Hospital, Karachi, as of April 2017, only four therapists have been certified by VitalStim. This phenomenon is mainly attributed to the following: low trust in the effectiveness of therapy, lack of trained professionals, and lack of resources, whether professional or personal. These misconceptions and barriers also affect evidence-based clinical decisions and treatment outcomes. [109].

The Ampcare ESP system uses NMES with electrodes placed under the chin and is designed to target the suprahyoid muscle group [110]. This particular electrode layout departed from the approach employed in previous studies, and the design was based on the work of Burnett et al. [111], 2003, which aimed to identify the muscle groups most closely associated with laryngeal elevation function.

### 6.2.2 rPMS

The limitation of PMS compared to NMES is that its larger coil makes it difficult to stimulate the suprahyoid muscle precisely. However, the latest rPMS technology has developed smaller coils to overcome this problem. rPMS is superior to NMES in elevating the hyoid bone at rest, and patients can drink 10 ml of liquid almost normally when rPMS is performed [100]. Two weeks of rPMS treatment with 5400 pulses per day significantly increased the strength of the suprahyoid muscle, which appeared to be superior to that of conventional head lifting exercises [112]. Current rPMS systems, such as Pathleader™ from the IFG, Sendai, Japan, deliver 1800 pulses at 30 Hz in 2 minutes, showing feasibility for patients with dysphagia [113]. In addition, a single 10-minute treatment (1200 pulses) with an MMC-90 parabolic coil improved swallowing speed and ability in stroke patients [92].

## 7. Technological Advancements and Future Directions

Recent technological advancements have significantly impacted the management of sarcopenic dysphagia.

### 7.1 Impact of recent technologies in sarcopenic dysphagia management

Currently, the management of sarcopenic dysphagia presents a complex challenge, necessitating a comprehensive strategy that integrates various therapeutic modalities. With the advancement of research, an increasing array of novel technologies has been progressively emerging, offering renewed hope for elderly patients afflicted with sarcopenic dysphagia. This development signifies a promising direction for enhancing the quality of care and outcomes for this patient population.

#### 7.1.1 Robotic swallowing technology

With the development of technology, swallowing robot technology has become an innovative treatment method. With the development of technology, swallowing robot technology has become an innovative treatment method. This technique helps patients reestablish their swallowing ability by simulating muscle activity during normal swallowing. Although this is a relatively new field, preliminary findings are encouraging and suggest that swallowing robotics has the potential to become an essential treatment for sarcopenic dysphagia in the future.

The soft robotic tongue is a soft robotic actuator inspired by the human tongue. The wettability properties of the soft robotic tongue are designed to mimic the natural lubrication mechanisms in the oral cavity, providing a unique tool for studying oral lubrication and its effects on related diseases such as xerostomia. However, in the absence of lubricant, a slight increase in residue can be observed, but there is little effect on the transit time of food. This discovery promotes further studies to consider lubricating fluid models more in line with real situations to simulate the role of salivary membranes more precisely.

At the same time, the flexible robotic tongue can apply physiologically reasonable fluid pressure during swallowing. *In vitro* swallowing tests showed a corresponding increase in palatal pressure when increasing bolus viscosity and applying higher pressure on the anterior part of the tongue under applied pressure.

Due to the high degree of freedom and fine control of actuators, the effects of poor tongue coordination on the swallowing process can be investigated for the possibility of new rheological properties to mitigate these effects and, consequently, achieve more stable food propulsion and enhanced swallowing safety. This technique helps patients reestablish their swallowing ability by simulating muscle activity during normal swallowing. Although this is a relatively new field, preliminary findings are encouraging and suggest that swallowing robotics has the potential to become an essential treatment for sarcopenic dysphagia in the future [114].

In addition, current transoral robotic technology has demonstrated a role in the treatment of swallowing dysfunction caused by oropharyngeal cancer [115]. Compared with traditional procedures such as mandibulotomy, tracheotomy, and free flap reconstruction, robotic surgery is more flexible, and the use of transoral robots for the same surgical purpose allows for the completion of more difficult dissections, contributing to the improvement of the operator's surgical skills as well as the minimization of the negative effects of surgery on the patient [116].

Due to the limitations of the current study, such as the lack of long-term follow-up data and economic benefit analysis, future research should still focus on evaluating the long-term effects and economic feasibility of such robotic procedures to better guide clinical practice [116]. At the same time, clinicians need to carefully consider the advantages and disadvantages of each treatment method when choosing a treatment and formulate a personalized treatment plan for patients with multidisciplinary team cooperation. This will not only improve the effectiveness of treatment, but also maintain the quality of life of patients to the greatest extent [117]. Future studies should aim to further optimize treatment options, reduce treatment-related functional impairment, and provide patients with more personalized and precise treatment regimens [115].

#### 7.1.2 Stem cell treatments

Dysphagia, a condition with a significant prevalence yet considerable treatment challenges, often encounters limitations within the scope of current medical interventions. Traditional therapeutic approaches frequently fall short of expectations, particularly in patients who lack native functional tissues. In these patients, novel therapies such as stem cell implantation may represent a new avenue to improve swallowing function. In this context, stem cell implantation has emerged as a promising new strategy for enhancing swallowing function [118, 119]. Stem cell therapy targets critical functions such as regenerating damaged tissues, improving muscle and nerve function, and restoring salivary gland activity, providing hope for treating diseases currently limited by traditional treatments. Although research in this field is still in its early stages, preliminary results show the great potential of using stem cells to treat oropharyngeal dysphagia, which undoubtedly deserves further in-depth study [120]. Several studies have shown that stem cell therapy can improve various causes of sarcopenic dysphagia.

##### Laryngeal muscle dysfunction

Stem cell therapy has shown promise in laryngeal muscle regeneration, especially in the case of vocal cord paralysis due to nerve damage. In one study, investigators injected autologous muscle-derived stem cells (MDSCs) into the thyroarytenoid muscle of rats after unilateral recurrent laryngeal nerve denervation. This increases muscle volume and diameter, and some animals exhibit weak vocal fold adduction [121]. These improvements were attributed to muscle augmentation rather than reinnervation, suggesting that stem cell therapy can restore laryngeal muscle function and potentially prevent aspiration [121].

##### Tongue Dysfunction

Stem cell therapy targeting patients after head and neck cancer treatment, including the use of muscle stem cells and collagen-rich hydrogels, aims to improve tongue function. This treatment strategy not only promoted an increase in tongue weight but also confirmed the regeneration of muscle tissue by detecting desmin-positive cells [122, 123]. In addition, the formation of new blood vessels and the preliminary phenomenon of neuralization indicate the possibility of restoring functional swallowing ability [122].

##### Xerostomia

The main goal of stem cell therapy for treating xerostomia is to restore the function of the salivary glands. Transplantation of cultured salivary gland stem cells into irradiated salivary glands significantly increased salivary gland production and the acinar cell surface area [124, 125]. This development highlights the critical potential of stem cell therapy to alleviate symptoms of dry mouth and improve swallowing function.

##### Cricopharyngeal dysfunction

In treating cervical pharyngeal dysfunction, the combination of stem cell therapy and cervical pharyngeal myotomy has shown the potential to improve the function of the upper esophageal sphincter (UES) [126]. Patients empirically supported this treatment strategy's effectiveness in terms of subjective feelings of improvement in swallowing function and reduction in swallowing time [126].

##### Poststroke Swallowing Difficulties

In the field of poststroke dysphagia treatment, research on stem cell therapy has focused on the recovery of neural coordination and muscle function. Although current research focuses on promoting neuronal regeneration and optimizing brain structure, translating these scientific achievements into practical improvements in swallowing function needs to be solved. Given the complexity of the swallowing mechanism, further exploration is needed to clarify how stem cell therapy can effectively promote the recovery of swallowing function in stroke patients [127-131].

##### Esophageal Repair

Stem cell therapy has shown significant potential in the recovery of esophageal function, especially for treating esophageal motility disorders, achalasia, and gastroesophageal reflux disease (GERD) [132, 133]. Although research on stem cell therapy for these diseases is relatively limited, its possibilities have been explored. Dysphagia caused by the esophagus has a variety of etiologies, which poses a challenge in identifying targeted treatment options. However, stem cell therapy has opened new possibilities for treating these primary esophageal diseases that affect swallowing function, providing new directions for future treatment.

At present, most of the research on stem cells for the treatment of dysphagia is still in the early stage, but these studies show the initial efficacy of stem cells in restoring structure and function. With further research, stem cell therapy is expected to become a new approach for the treatment of refractory dysphagia, especially in patients with limited efficacy of traditional therapies [120].

These novel treatments, ranging from electrical stimulation to stem cell therapy, have shown great potential for improving swallowing function and quality of life. Although more clinical trials are still needed to verify the efficacy and safety of these approaches, they provide valuable directions for future treatment.

### 7.2 Potential areas for future research

In the future, sarcopenia screening and early detection of risk factors may become more critical, and the fundamental skeletal muscle genotype may play a pivotal role in tailoring precise therapeutic interventions for sarcopenia [67]. Understanding and incorporating the genetic underpinnings of skeletal muscle in treatment strategies are crucial for enhancing the efficacy of interventions targeted at addressing sarcopenia. This recognition of the significance of the skeletal muscle genotype underscores the need for a nuanced and personalized approach to managing sarcopenia, aligning with the principles of precision medicine. Furthermore, the acknowledgment of genetic factors adds a layer of complexity that necessitates thorough consideration in developing and implementing interventions, contributing to advancing targeted and effective strategies for sarcopenia treatment. For example, certain radiomic assays can be used as potential biomarkers in patients with sarcopenia, especially in cancer patients. Radiomics data on skeletal muscles for the diagnosis of sarcopenia have been previously obtained in patients diagnosed with non-small-cell bronchial carcinoma. The findings from this analysis contribute to the broader understanding of the applicability and potential challenges associated with employing radiomics data in the context of sarcopenia diagnosis, offering insights that could be valuable for future research and clinical applications [134, 135].

Sarcopenia is a vital component of dystrophic dysphagia. Along this line of thinking, precision treatment interventions in the field of dystrophic dysphagia can be explored. For example, CT is crucial for more diagnoses and treatment procedures. We can obtain essential data on the relevant muscles of patients with sarcopenic dysphagia through CT, artificial intelligence technology, and extensive data analysis, improving the ability of imaging reports to improve the diagnosis rate of sarcopenic dysphagia in the population. For example, genotype analysis of patients with diagnosed sarcopenic dysphagia can help to provide support for personalized treatment and targeted rehabilitation guidance for patients with sarcopenic dysphagia.

## 8. Discussion

Sarcopenic dysphagia is a condition with a poor prognosis and serious complications that are more prevalent and easily overlooked in the elderly population. With the current aging society in full swing, the resulting social burden will likely increase.

Management of dysphagia requires a multidisciplinary, patient-centered approach. Because of the lack of concise guidelines, goals of care, complex medical histories, and frailty, as well as each patient presenting with varying needs, the management is complex [136]. In the current extensive literature review, we found that the tools used to diagnose sarcopenic dysphagia in different countries and regions are inconsistent, heterogeneous, and lack validation of objective measures of swallowing dysfunction and that the use of different diagnostic criteria predisposes patients to differences in the prevalence of the disease. More research is needed to validate these sarcopenic dysphagia diagnostic tools, establish thresholds in various populations, and study their usefulness as screening tools for dysphagia and swallowing dysfunction. We encourage the development of sarcopenic dysphagia diagnostic tools and algorithms that are easy to use and that facilitate sarcopenic dysphagia screening and diagnosis.

Sarcopenic dysphagia has an important impact on swallowing function, Activities of Daily Living (ADL) and life prognosis of patients. The combination of rehabilitation, nutrition and oral management may be beneficial for patients with dysphagia. Rehabilitation nutrition nursing is a key point in the recovery process of patients with sarcopenia. Each link needs the cooperation and team implementation of multidisciplinary team professionals, and its goal setting should be specific, measurable, achievable, relevant and time limited. Because the current diagnostic tools for sarcopenia are not uniform, and the objective measures of swallowing dysfunction are less verified. Future review of diagnostic criteria and development of clinical practice guidelines for sarcopenic dysphagia are needed [137].

By designing a simple screening and protection process to assess the risk of screening and preventing dysphagia in elderly patients, it can effectively improve the recognition rate of dysphagia and reduce the occurrence of complications through early intervention. At the same time, simple screening and prevention are easy to promote in routine care and are expected to be extended to outpatient care and home care in the future. By applying it in different nursing settings, especially for patients with sarcopenic dysphagia in the geriatric ward, it fills an important gap in the current nursing process. It is worth noting that special attention needs to be paid to the satisfaction of the nursing team in implementing the procedure to ensure that standardized practice is maintained, and the risk of dysphagia is reduced despite high turnover. Through multidisciplinary cooperation and the use of standardized tools, we provide new ideas for improving the quality of life and nursing effect of elderly patients [64].

With the advancement of medicine and technology, an increasing number of new techniques and tools are emerging, and artificial intelligence (AI) is set to revolutionize dysphagia management, including the treatment of sarcopenic dysphagia in the future [138]. Despite the continuous emergence of new technologies, their promotion and application face multiple challenges. Through this review, we hope to attract more clinical and researchers' attention to this phenomenon in the future and promote the promotion and application of more emerging diagnostic and therapeutic technologies in developing countries such as Pakistan [109].

We hope that in the future when dysphagia with no apparent underlying cause occurs during or after hospitalization, the corresponding screening assessment or imaging methods can promptly identify sarcopenic dysphagia and take early measures to intervene through the formulation of individualized dietary and nutritional support programs. And we encourage patients to engage in appropriate activities and carry out targeted rehabilitation exercises and treatments to reduce the related complications and improve the prognosis and quality of life of elderly people.
